# Continuous electrical stimulation of the dorsal root ganglion (drg-s) as a salvation therapy in patients previously treated with spinal cord stimulation. systematic review and pooled analysis

**DOI:** 10.1007/s10143-025-03769-7

**Published:** 2025-09-22

**Authors:** Juan Carlos Acevedo-Gonzalez, Isabella Lacouture-Silgado

**Affiliations:** 1 Departamento de Neurociencias, Facultad de Medicina, Facultad de Medicina, Pontificia Universidad Javeriana, Bogotá, Colombia; 2 Estudiante de Medicina XII semestre, Facultad de Medicina, Pontifician Universidad Javeriana, Bogotá, Colombia; 3 Unidad de Neurocirugía, Hospital Universitario San Ignacio, Bogotá, Colombia

**Keywords:** Dorsal root ganglion, Neuromodulation, Salvage, Habituation, Spinal cord stimulation, Salvation

## Abstract

**Background context:**

Treatment techniques on the dorsal root ganglion has offered a broad overview in the chronic pain. The aim is to review the existing evidence on DRG-s as a “salvation” of spinal cord stimulation therapies. We make a grouped analysis of the patients treated.

**Methods:**

A systematic search of the medical literature was conducted based on the principles recommended by PRISMA. In the phase 1 (DRG-S therapy as a “salvation” treatment for patients with SCS) the following search words were used: “ganglia”, “DRG”, “dorsal root ganglion”, “neurostimulation”, “salvage”, “salvation”, “habituation”, “spinal cord stimulation”. In the phase 2 studies using DRG-S therapy with previous SCS were included. The following words were used as search words in the databases: “spinal ganglia”, “DRG”, “dorsal root ganglion”, “neurostimulation”. The search included articles from each databases creation through August 2025. Inclusion: Systematic review, randomized clinical trials, observational studies, case series. Cadaveric and experimental articles were excluded.

**Results:**

In the phase 1, 230 articles were identified and 10 of them were selected for analysis. In the phase 2, 530 articles were identified and 45 of them were selected for analysis. The Prisma checklist for systematic reviews was applied and the risk of bias and the quality of the study were evaluated based on the STROBE and CONSORT criteria. 147 patients were identified has having previously had a SCS, who had previously undergone a SCS trial phase or who had an implanted and active system at the time of the study. In 31/147 patients, detailed information on clinical or therapeutic aspects related to the SCS was not included in the articles. The cause of chronic pain was most frequently reported as CRPS (37%) and PSPS (36%). It included other pathologies such as: chronic pelvic pain, radiculopathy, peripheral neuropathic pain, gonalgia, post-thoracotomy pain, post-inguinal herniorrhaphy pain, phantom limb pain and severe peripheral artery disease. The follow-up period and the analysis of the results were very varied, but it can be concluded that in most cases the use of DRG-s was indicated due to a poor clinical response to SCS despite not specifying what type of stimulation was being performed (in most cases SCS-t) or whether the therapy was salvaged with other forms of SCS (Burst, high frequency, ECAP- controlled closed-loop, Differential Target Multiplexed,etc.). In most patients implanted with DRG-s the clinical result was better and the degree of patient satisfaction with the new therapy was clear.

**Conclusions:**

DRG-s is a useful procedure in the treatment of chronic pain. It emerges as a complementary tool that can be used even in patients who have (or have had) an SCS. It should be included together with new forms of spinal cord stimulation in the therapeutic arsenal of patients with refractory chronic pain. There will be situations where DRG-s will help improve patients with loss of SCS efficacy, just as the opposite may also occur. A detailed clinical analysis will always be necessary to ensure the benefit of the patient and the sustainability of healthcare systems.

## Introduction

Since 1967, when the first implantation of a spinal cord stimulation system (SCS) was made, the use of electricity has played a very important role in the control of chronic pain [1]. New technologies and improvement of the technique have made it possible to improve clinical results and popularize its use [2, 3]. Even advances have recently been introduced in the SCS pattern with promising effects (Burst, high frequency, ECAP- controlled closed-loop and Differential Target Multiplexed) [4–7]. However, there are still patients who do not respond to spinal cord stimulation even with the use of these new technologies [2]. In parallel, in the last 10 years and from the classic concepts of Patrick Wall and Marshall Devor, continuous stimulation of the dorsal root ganglion (DRG-s) arises, positioning itself with new and better indications in CRPS, chronic distal pain in the extremities, pelvic pain, somatic pain, etc. [8, 9]. Its multifactorial mechanism of action is directed towards the DRG neurons, the sandwich synapses and especially at the microglia cells that form independent neuron-glia-neuron action units with a retrograde action on receptors, sympathetic fibers (white and gray communicating branches) and peripheral nociceptors, an anterograde action towards the spinal cord, posterior cords, wide-range neurons (WDR), spinal inhibitory interneurons and ascending/descending pathways [10]. With initially very specific indications, its therapeutic range of action (DRG-s) in painful pathologies and in chronic pain has been extended too much, even being considered as the main rescue (salvation) of patients who do not respond to SCS, including patients without a definitive implant with a negative SCS test phase and patients with SCS who develop habituation phenomena to the generally tonic therapy (SCS-t) [10, 11]. The recent rescue management guidelines published by the International Neuromodulation Society serve as a context for this systematic review and pooled analysis that seeks to collect the available data on patients in whom DRG-s was used as a rescue of a previous SCS [6].

## Materials and method

### Literature review

- Phase 1 (DRG-S therapy as a salvation treatment for patients with SCS) A systematic search of the medical literature was conducted based on the principles recommended by PRISMA (Preferred Reporting Items for Systematic Reviews and Meta-analysis). The following search words were used in the databases: “ganglia”, “DRG”, “dorsal root ganglion”, “neurostimulation”, “salvage”, “salvation”, “habituation”, “spinal cord stimulation”. Logical connectors such as “and”, “not” and “or” were used. The following databases were reviewed: Pubmed, Scopus, Medline, web of science and Embase. The search included articles from each databases creation through July 2025 and was specifically oriented towards those studies in which DRG-S therapy was used as a life-saving treatment for patients with SCS.
- In the phase 2 studies using DRG-S therapy with previous SCS were included. A ​​systematic search of the medical literature was conducted based on the principles recommended by PRISMA (Preferred Reporting Items for Systematic Reviews and Meta-analysis). The following words were used as search words in the databases: “spinal ganglia”, “DRG”, “dorsal root ganglion”, “neurostimulation”. Logical connectors such as “and”, “not” and “or” were used. The following databases were reviewed: Pubmed, Scopus, Medline, web of science and Embase. The search included articles from each databases creation through July 2025 and was oriented to all clinical studies that used DRG-S therapy to search their databases to determine whether they included patients who previously had an SCS.

The “Rayyan” program was used to collect the information and facilitate the analysis process. This application serves a platform that allows easy manipulation of the information obtained. Each of the authors independently reviewed the summary of each of the articles found and applied the following criteria in both phases. Inclusion: Systematic review of the literature, randomized clinical trials, observational studies, case series. Exclusion: Cadaveric and experimental articles.

### Selection of articles phase 1 (specific for salvage)

**Fig. 1:**
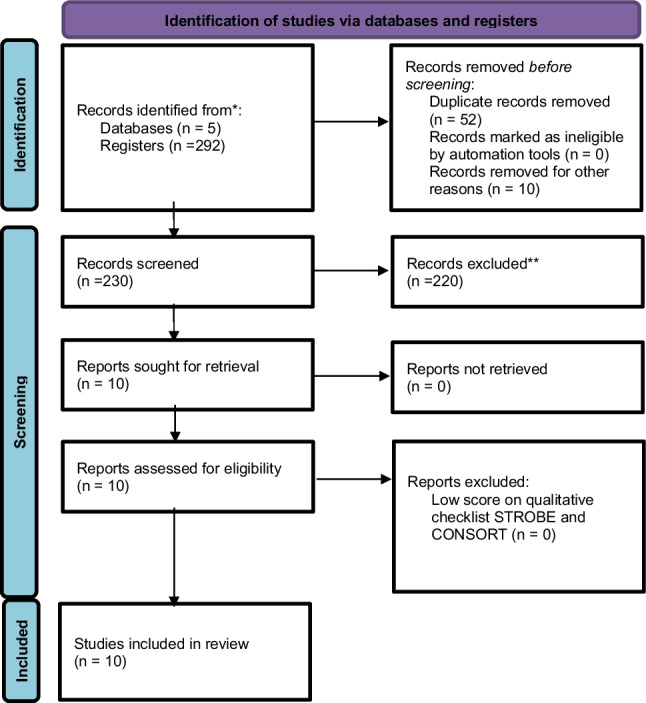
PRISMA. In the phase 1 (DRG-S therapy as a “salvation” treatment for patients with SCS)

There were 230 articles identified in the consulted databases (after eliminating duplicates). The selection criteria (Inclusion/Exclusion) were applied based on the independent reading of the abstracts by each of the authors on the Rayyan platform and 220 articles were excluded. The remaining 10 selected articles were reviewed in full by each of the authors and included in the analysis. Epidemiological, methodological and data related to the results found were entered into an Excel spreadsheet. The Prisma checklist for systematic reviews was applied and in each of the articles the risk of bias and the quality of the study were evaluated based on the STROBE and CONSORT criteria. (Fig. 1-PRISMA phase 1, Table 1-Strobe phase 1, Table 2-Consort-phase 1) [11–21].

**Table 1.**
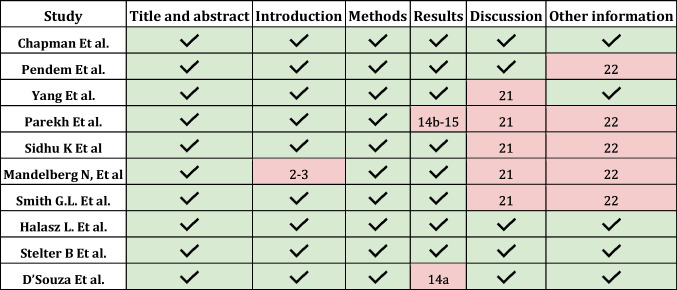
Analysis of STROBE in Phase 1 Studies: The blue boxes were the ones that fully met the criteria evaluated. The pink boxes were the ones that partially met the criteria evaluated, indicating the item that was considered incomplete.

**Table 2.**
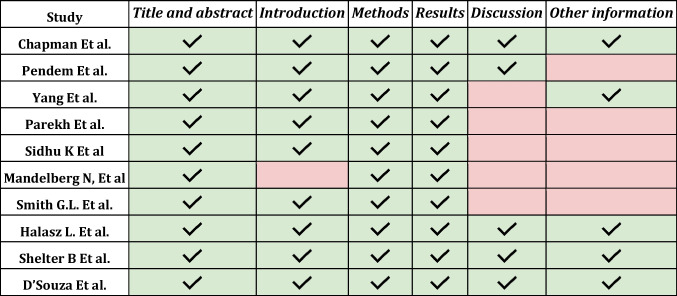
Analysis of CONSORT in Phase 1 Studies. The blue boxes were the ones that fully met the criteria evaluated. The pink boxes were the ones that partially met the criteria evaluated.

**Table 3:** This data is mandatory. Please provide

Author	Reference	Type of study	Patients (#) salvation	Summary and demographic characteristics	Description 1	Description 2	Description 3
Chapman – 2022 (Phase 1)	Neurom2022;25(7):1024–1032	Retrospective study	60	56 + −12 years./34 m and 26 w/24 CRPS, 24 Failed back. 12 (pelvic pain, radicular pain, low back pain, peripheral neuropathy, knee pain)/32 had SCS-t, 18 Burst, 10 high frequency./34-month DRG-s follow-up/VAS went from 8.7 + −1.2 to 3.8 + −2.1. They included patients with a negative SCS test phase (less than 50% improvement) and patients with habituation./ODI was evaluated and opioid consumption was evaluated./all DRG-s had a trial phase but 5 were intraoperative./opioid consumption went from 23 mg on average to 15 mg on average. DRG-s serves as rescue in 90% of patients (56/60)	1patient withdrew from the study due to unrelated malignancy	2 patients did not approve the new stimulator	2patients with DRG-s were explanted 21 and 28 months later due to lack of improvement
Mandelberg −2022 (Phase 1)	Neurosurgery 2022;68(Supp_1):56	Retrospective study	-	It is the presentation of Chapman's series in the 2021 Annual Meeting of the Congress of Neurological Surgeons: Austin, Texas October 16–20, 2021	-	-	-
Shelter – 2021 (Phase 1)	Neurom 2021, 24(4): 622–33	Systematic Review	-	Systematic review for chronic pain treatment (different from SDRC) with DRG-S. The search was conducted in 2020 (December 1999—2020) 1881 studies were found and 28 studies were selected, with 354 patients. Among those 28 articles found, five studies describe the lack of efficacy of a SCS prior to the implementation of DRG-S (Hunter-2017, Chapman-2020, Chapman −2020, Hunter-2019 and Giordano-2018)	-	-	-
D¨souza −2022 (Phase 1)	Adv Ther 2022. 39(10):4440–4473	Systematic Review	-	Systematic search from the beginning of the databases until March 2022 of clinical studies in patients treated with DRG-S for neuropathic pain of lower limbs. He found 149 articles and selected 40. Among these studies they mention five of them in which they describe patients who had previously been treated with SCS (Van Bussel-2018, Chapman-2017, Goebel-2018, Levy-2020, Ghosh-2021)	-	-	-
Yang 2017 (Phase 1)	Neurom 2017;20(7):703–707	Case series	2	-	P1: 43a/M/CRPS in MID due to crush injury in 2014/SCS Boston/1 lead/16 contacts in T8/improvement 3 months/DRG-s trial phase 90% improvement/follow-up 8 months improvements	P2: 50 a/CRPS in both MsIs/crush injury in both legs 2007/2012 SCS st Jude/Octrode T9 + EON/in 2015 0% improvement/HYBRID: without removing the SCS they put DRG-S in bilateral L3/they tried both interleaved/greater improvement with DRG-S	-
Halasz– 2021 (phase 1)	Stereotactic and functional Neurosurgery 2021; 99 (suppl 1): 120–21. Poster	Case series	2(6)	3 patients neuropathic low back pain (2 of these had been implanted with SCS without improvement)/1 p T5 level after tumor resection/1p post-thoracotomy pain/1 ilioinguinal pain after inguinal hernia surgery/All trial phase 3 weeks/mean age 57.83 + −8.9/evolution with pain 9.8 +—7 years./Of the 6, only 2 were already implanted	The two patients who were already implanted with SCS-t suffered from Low back pain	Overall result: VAS preop 7.83 + −1.17 and move to 1.50 +—1.05	There is no more information because it is an abstract of a poster
Smith – 2021 (Phase 1)	Neurom2021; 24(4):763–768	Case series	2(3)	It is a study that presents 3 patients in which there was difficulty placing DRG-s using the traditional technique and proposes TRANSGRADE technique (from top to bottom). Among the three patients, 2 already had SCS. They only mention that they had already had SCS and it had been removed	P1: 35 years/CRPS right ankle/DRG-s in L5 displacement of pain a year later/DRG-s S1 and L4 were added. This required accommodation of the electrode that was displaced	P2: 45 years/sx postlaminectomy/radiculopathy l5 and s1 left/SCS-t/no improvement with test phase/test then DRG-s/50% improvement/drg-s L5 implant 80% improvement	P3:57 years/Causalgia + neuropathic pain/secondary to accident felling trees + low back pain/SCS-t/no improvement/DRG-s test at t12 and l5 improvement greater than 50%
Pendem – 2021 (Phase 1)	Cureus2021;13(1):e12713	Case series	1	1 patient	P1: 60 years/man/CRPS in right leg Budapest criteria/traffic accident rupture of posterior tibial tendon//	Previous SCS-Boston 16 contact in upper part of T8/improvement in pain by 80% for 4 months/worsening	Relapse of pain/DRG-s in L5 and S1/improvement greater than 90%/discontinued opioids
Sidhu – 2022 (phase 1)	Neurom 2022;25(7):S92	Case series	3	3 patients with DRG-s after SCS to treat painful neuropathy	P1: Chronic neuropathic right knee pain/5 reviews of SCS for poor effectiveness./33% improvement/improvement after DRG-s	P2: CRPS MII/SCS very limited time to improvement/50% improvement with DRG-s	P3: Chronic neuropathic pain in the left abdomen/36% improvement with SCS/Improvement of more than 50% with DRG-s
Parekh-2022 (Phase 1)	Neurom 2023;26(4):S218	Oral presentation	1	1 patient	P1: 30 years/woman/Sx postlaminectomy/rescue with DRG-s T12 and S1	Previously had SCS-t/Had 50% improvement for 1 year after SCS-t then failure/SCS was removed and DRG test phase was done -s T12 and S1	Day 7 improvement of 90%/does not mention follow-up. I had low back pain + neuropathic pain in MsiS/
Huygen-2019 (Phase 2)	Neurom 2020. 23(2);213–221	Prospective clinical studies	-	No information was found related to patients treated with DRG-s and who had previously had SCS	-	-	-
Kalleward-2019 (Phase 2)	Pain Pract. 2019 Feb;19(2):204–210	Prospective clinical studies	-	No information was found related to patients treated with DRG-s and who had previously had SCS	-	-	-
Kalleward-2020 (Phase 2)	Neurom. 2020 Feb;23(2):196–202	Prospective clinical studies	-	No information was found related to patients treated with DRG-s and who had previously had SCS	-	-	-
Levy-2019 (Phase 2)	J Pain. 2020 Mar-Apr;21(3–4):399–408	Prospective clinical studies	-	No information was found related to patients treated with DRG-s and who had previously had SCS	-	-	-
Morgalla-2019 (Phase 2)	Neurom 2019;22(1);36–43	Prospective clinical studies	-	No information was found related to patients treated with DRG-s and who had previously had SCS	-	-	-
Chapman −2021 (Phase 2)	Pain Pract. 2021 Jun;21(5):568–577	Prospective clinical studies	-	No information was found related to patients treated with DRG-s and who had previously had SCS	-	-	-
Sverrisdottir −2020 (Phase 2)	JACC Basic Transl Sci. 2020 Sep 16;5(10):973–985	Prospective clinical studies	-	No information was found related to patients treated with DRG-s and who had previously had SCS	-	-	-
Lo Bianco-2020 (Phase 2)	Neurom. 2021 Jun;24(4):774–778	Prospective clinical studies	-	No information was found related to patients treated with DRG-s and who had previously had SCS	-	-	-
Gravius-2019 (Phase 2)	Neurom. 2019 Jan;22(1):44–52	Prospective clinical studies	-	No information was found related to patients treated with DRG-s and who had previously had SCS	-	-	-
Han −2024 (Phase 2)	Front Neurol. 2024 Apr 10;15:1,366,796	Prospective clinical studies	-	No information was found related to patients treated with DRG-s and who had previously had SCS	-	-	-
Sankarasubramanian – 2021 (Phase 2)	Neurom. 2021 Jun;24(4):672–684	Prospective clinical studies	-	No information was found related to patients treated with DRG-s and who had previously had SCS	-	-	-
Weising – 2022 (Phase 2)	Neurom 2022;25(7):998–1005	Prospective clinical studies	4 (30)	There were 30 patients admitted to the study, of which only 22 completed follow-up. Exclusion criteria included having an SCS-s, but 4(30) had used an SCS-t at some point in their medical history and were therefore included in the study	At the last follow-up, only 16 had DRG-s implanted	It only states that 15% of the patients (4/30) had a history of using neuromodulation but does not describe anything about them	-
Eldebe-2022 (Phase 2)	Pain 2022; 163(4): 702–715	Prospective clinical studies	2 (32)	There were 32 patients implanted with DRG-s and followed up for 7 years. In that period, he points out that 5 patients had to be explanted and had an SCS implanted	There are no details about those 2 patients	The interest of this article is that it mentions 2 patients in whom SCS became the salvation of a DRG-s that did not work	-
Mol-2022 (Phase 2)	Neuromod. 2023 Dec;26(8):1788–1794	Prospective clinical studies	-	No information was found related to patients treated with DRG-s and who had previously had SCS	-	-	-
Piedade-2022 (Phase 2)	Neurochir 2022. 164(4):1193.1199	Prospective clinical studies	-	No information was found related to patients treated with DRG-s and who had previously had SCS	-	-	-
Piedade-2023 (Phase 2)	Neurom. 2019 Dec;22(8):951–955	Prospective clinical studies	-	No information was found related to patients treated with DRG-s and who had previously had SCS	-	-	-
Chapman-2024 (Phase 2)	Neurom. 2024 Jul;27(5):881–886	Prospective clinical studies	-	No information was found related to patients treated with DRG-s and who had previously had SCS	-	-	-
Schultheis-2024 (Phase 2)	Neurom. 2024 Jan;27(1):151–159	Prospective clinical studies	-	No information was found related to patients treated with DRG-s and who had previously had SCS	-	-	-
Mons-2024 (Phase 2)	Neurom. 2024 Jan;27(1):172–177	Prospective clinical studies	-	No information was found related to patients treated with DRG-s and who had previously had SCS	-	-	-
Rigoard-2025 (Phase 2)	Neurom. 2025 Feb;28(2):283–296	Prospective clinical studies	-	No information was found related to patients treated with DRG-s and who had previously had SCS	-	-	-
Van Bussel – 2018 (phase 2)	Pain Practice 2018;18(1):87–93	Prospective clinical study	12	Compares the two stimulation methods in random order, in patients with CRPS of the knee. They underwent a trial phase of SCS and then DRG-s for 16 days, the patient was asked which was better. Among the exclusion criteria was previous stimulation. SCS Medtronic – 8 electrodes. DRG-s two 4-contact electrodes. Both methods were implemented in the second surgical procedure. First week, the first stimulation was done, which was randomized, a 2-day stimulation wash was done. After the testing phase, the cable of the least effective system was removed and the one that served the generator was connected with a 12-month follow-up. Initially there were 74 p but some were excluded due to an alteration in the diagnosis or because they had had SCS	11 women/1 man/mean age 38.7 years/initial VAS 68./in 5 patients both methods were successful	10 p (83.3%) preferred DRG-s and 2p (16.7%) SCS	-
Hunter-2019 (Phase 2)	Neurom. 2019 Jan;22(1):87–95	Retrospective study	-	No information was found related to patients treated with DRG-s and who had previously had SCS	-	-	-
Verrills – 2019 (Phase 2)	Neurom2019; 22(8):937–42	Retrospective study	18	Inclusion criteria include having failed neuromodulation. The aim of the study is to compare DRG-s with paresthesias and without paresthesias They conclude that even without paresthesias, DRG-s is useful	There is no information regarding the included patients who had SCS	-	-
Martin-2020 (Phase 2)	Neurom. 2020 Feb;23(2):245–251	Retrospective study	-	No information was found related to patients treated with DRG-s and who had previously had SCS	-	-	-
Martin-2020 (Phase 2)	World Neurosurg. 2020 Nov;143:e303-e308	Retrospective study	-	No information was found related to patients treated with DRG-s and who had previously had SCS	-	-	-
Hogedorn-2021 (Phase 2)	Neurom. 2021 Jun;24(4):695–699	Retrospective study	-	No information was found related to patients treated with DRG-s and who had previously had SCS	-	-	-
Hines-2022 (Phase 2)	Neurom. 2022 Oct;25(7):1040–1044	Retrospective study	-	No information was found related to patients treated with DRG-s and who had previously had SCS	-	-	-
Graca- 2023 (Phase 2)	Neuromo 2023; 26(8):1781–87	Retrospective study	1	Of the 20 patients implanted with DRG stimulation, only one was described as having previously received SCS. The objective of the study was to demonstrate la efficacy and safety in cervical or upper thoracic DRG-s in patients with MsSs CRPS	There is no information regarding the included patients who had SCS	-	-
Tabatabaei- 2024 (Phase 2)	Neurom 2024;27(1):141–50	Retrospective study	2 (13)	There were 13 implanted patients, among whom 2 patients had SCS. Bilateral DRG-s in Th12 for back pain	P1: 45 years/man/PSPS2/I improve with DRG-s	P2: 60 years/woman/PSPS2/improved with DRG-s	There is no specific information regarding the included patients who had SCS
Mullins – 2024 (Phase 2)	Pain Medicine 2024; 25(2):116–124	Retrospective study	20	Patients treated synchronously with DRG-s + SCS. 20 p implanted for Neuropathic pain. 26 p test phase. 20p successful test. 18p implanted (1 was lost and another discontinued therapy)	It is striking that the SCS is placed in t12-L1. They did not use specific DRG-s electrodes but put an SCS electrode as DRG-s	Patients were given the option to use: only DRG-s, only SCS, or use both. There were 17 who completed the follow-up	9 p preferred the SCS + DRG-s combination and obtained an improvement of 78.5%. 3 p preferred only SCS improved 75%. 5 p preferred DRG-s and had an improvement of 81.9%
Fallowski – 2019 (Phase 2)	Neurom 2019; 22(1):96–100	Case series	2 (8)	8 implanted patients among whom 2 p had SCS. Dx peripheral neuropathy in legs. These two patients had the system implanted and although its effect was limited, they were not removed and they continued using the two systems.The average pain went from 7.4 to 1.5 at the last follow-up	-	-	There is no information regarding the included patients who had SCS
Hunter – 2019 (Phase 2)	Neurom 2019;22(1):87–95	Case series	2(7)	Pelvic pain. PPC. Treated with bilateral DRG-s trial L1 and S1. Only 4 were permanently implanted	P1: 63 years/woman/PPC after infection with sacral nerve stimulator to treat foot pain/bilateral plantar fasciitis/SCS in bilateral S1 foramen/explant 6 months later due to infection/when removing SCS increased pain/7 years after DRG-s	P2: 47 years/woman/PPC after many surgeries for endometriosis/rectal fistula treated/internal and external vaginal pain/anus/did CRPS after epidural steroids/SCS implant ineffective test and presented foot drop/DRG-s 100% improvement	-
Skaribas −2019 (Phase 2)	Neurom. 2019 Jan;22(1):101–107	Case series	-	No information was found related to patients treated with DRG-s and who had previously had SCS	-	-	-
Verma-2024 (Phase 2)	Pain Pract. 2024 Mar;24(3):567–572	Case series	-	No information was found related to patients treated with DRG-s and who had previously had SCS	-	-	-
Yang-2024 (Phase 2)	A A Pract. 2024 Jun 21;18(6):e01804	Case series	-	No information was found related to patients treated with DRG-s and who had previously had SCS	-	-	-
Thalla-2024 (Phase 2)	Cureus 2024 16(2): e55043	Case series	-	No information was found related to patients treated with DRG-s and who had previously had SCS	-	-	-
Piedade – 2024 (Phase 2)	Acta Neurol Belga 2024; mayo 18	Case series	1	They present the clinical case of a patient implanted with double technology: SCS + DRG-s	45 years old/man/PSPS2/SCS + DRG-s was implanted and several phases were carried out. I improved more with only DRG-s or with both therapies	-	-
Lee-2022 (Phase 2)	Cureus 2022; 14(1): e21193	Case series	-	No information was found related to patients treated with DRG-s and who had previously had SCS	-	-	-
Hunter – 2017 (Phase 2)	Neurom 2017;20:708–718	Case series	1(3)	The objective of this study is to describe the different forms of pain in amputee patients and to conduct a rF stimulation study of the DRG evaluating response to stimulation, thinking of a DRG-s. Only one patient in the study had previous SCS	67 years old/man/PLP phantom limb pain/RLP residual limb pain/right foot/amputation done 18 years before/crush trauma in 1982/amputation in 1998	SCS – Boston/improvement in pain but gradual loss of effect	Performed stimulation of the DRG with rF and stimulation is obtained from the painful area./90% improvement during testing phase DRG-s
Chapman-2020 (phase 2)	Neurom2020;23:203–212	Case series	4	It seeks to evaluate a series of cases treated with DRG-s in Th12 for patients with low back pain. Among the description, it indicates patients who previously had SCS but does not describe them	There is no information regarding the included patients who had SCS	-	-
Chapman – 2020 (Phase 2)	A A Practice 2020;14(10):1–4	Case series	1(4)	They describe a modified implantation technique in 4 patients treated with DRG-s for PsPs2	P1: 56 years/woman/chronic low back pain and sacroiliac pain/3 lumbar surgeries with laminectomy t9-t11, decompression and fusion from L1 to S1	had failure in use of intrathecal morphine pump and in SCS which was explanted	DRG-s T12 and S1/60% improvement
Giordano- 2018 (phase 2)	Case report in anesthesiology 2018:5,832,401	Case series	1	No information was found related to patients treated with DRG-s and who had previously had SCS	37 years/chronic pain in the coccyx/onset of pain in 2009/coccyx fracture due to a fall from its own height/grade 10/10/pain in the area and radiating to both legs/Limitation in sitting and walking/	2011 SCS – ANS genesis saint jude, electrode placed through the sacral hiatus/still uses it/had infection at the surgical site but the equipment was not removed	DRG-s test phase 1/10/4 months later improvement greater than 90%/discontinued opioids
Goebel – 2018 (phase 2)	Pain Practice 2018;18(1):104–108	Clinical case	1	No information was found related to patients treated with DRG-s and who had previously had SCS	P1: Man/CRPS left foot after a soft tissue injury/	2010 SCS paddle/No stimulation of the pain area was achieved/Transtibial amputation in 2011/pain improvement	18 months later pain in stump/neuroma removed/postoperative CRPS/VAS 8/10/SCS test phase/pain area could not be covered/Then DRG-s test phase in left L4/after one month pain relief 25%./at 17 months 60% improvement
Ghosh – 2021 (phase 2)	Neurom 2021;24(4):769–773	Cases series	4	4 patients with CRPS in legs and SCS-t implanted. Poor coverage or incomplete coverage. DRG-s trial additional pain improvement greater than 60%. They didn't remove the SCS Patient 1: Woman/44 years/CRPS type 1 right upper limb and bilateral in legs/SCS-t./100% improvement for which she decided to implant SCS-t for right upper limb/Significant improvement but could not use the equipment at night due to uncomfortable paresthesias in legs./bilateral DRG-s l5 and s1/improvement 80% and 90% with both. 10-month follow-up	Patient 2: 30 years/woman/CRPS 1 in left leg after sprained ankle in 2005/multiple treatments including sympathetic blocks/SCS-t trial phase/pridigy abbot implant/partial improvement/poor pain coverage./VAS 8–10/10/DRG.s test phase/improvement in pain greater than 80% when using both	Patient 3: 28 years old/CRPS right leg, hit by vehicle 2014/sympathetic blocks/SCS-t improvement of more than 70%/habituation to improvement of only 20%/DRG.s/improvement of 70% with DRG alone and 90% with both therapies/13 months of follow-up	Patient 4: 52 years/Woman/CRPS type 1 in left leg/fall and multiple ankle fracture/SCS-t improvement of more than 50%/habituation/displacement of the percutaneous electrode/it was decided to implant a paddle/habituation up to 30% improvement only/DRG-s test phase improvement of more than 70% and with the two stimulators 80%
Chapman – 2021 (phase 2)	Annals of vascular surgery 2021; 74:519	Case series	1	P1: 55 years old/female/type 2 diabetes + peripheral arterial disease with ulceration of the first toe of the left foot/sympathetic block/medical treatment	trial phase of both SCS + DRG-s was dedicated/VAS 9/10/DRG- s L4 and L5 left + SCS T9T10	the DRG was activated first and washed for 3 days (off) and then SCS/improved to 1/10 with DRG, with SCS it improved to 3/10. oxygenation parameters were also higher for DRG-s	The insurance company did not authorize DRG-s and SCS was implanted./3-year follow-up improved and did not require amputation

### Selection of phase 2 articles: search for patients who underwent DRG-S salvage in general clinical studies (not specific to salvage) of DRG-S:

**Fig. 2:**
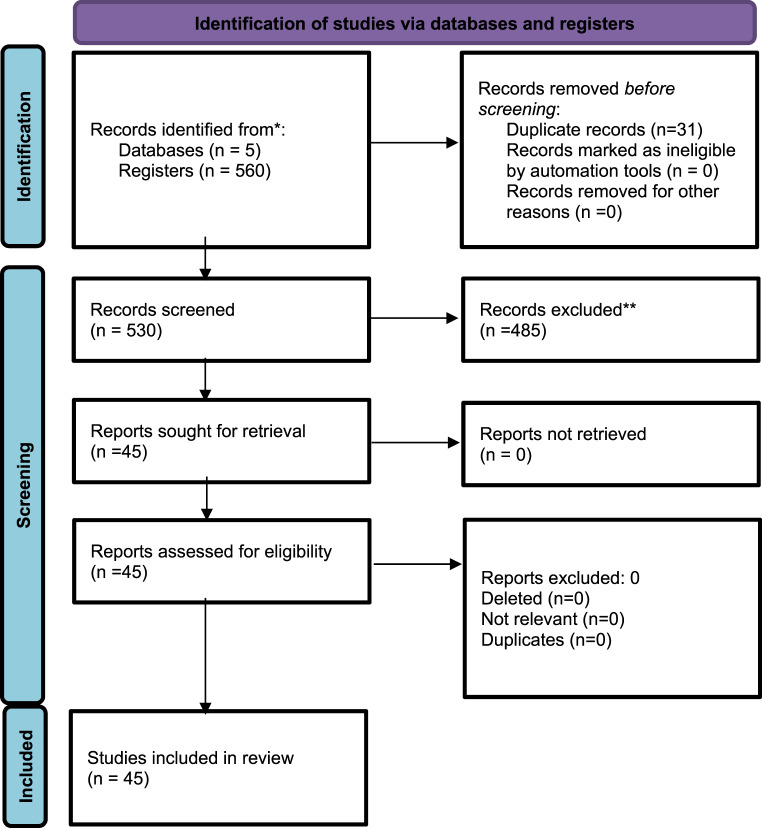
PRISMA. In the phase 2 (DRG-S therapy clinical studies)

A total of 530 articles were identified in the consulted databases (after removing duplicates). The selection criteria (Inclusion/Exclusion) were applied based on the independent reading of the abstracts by each of the authors on the Rayyan platform and 485 articles were excluded. The remaining 45 selected articles were reviewed in full by each of the authors and included in the analysis. Epidemiological, methodological and data related to the results found were entered into an Excel spreadsheet. (Fig. 2-Prisma phase 2) [2, 22–61].

## Results

### Classification and description of phase 1 articles

Eleven articles were selected, including 2 retrospective clinical studies (Chapman-2022 and Mandelberg-2022), 2 systematic reviews (Shelter-2021 and DSouza- 2022), 6 case series (Yang-2017, Halasz-2021, Smith – 2021, Pendem-2021, Sidhu-2022 and Parekh-2022) [11–21].

The two retrospective clinical studies were published in 2022 and use the same database [11, 12]. The Chapman study (2022) is a multicenter retrospective clinical analysis conducted between 2016 and 2020 presenting a series of 60 patients (age 56 + −12 years) who were implanted with a DRG-S system were presented after having failed SCS test phase (less than 50% improvement in pain) or habituation phenomena to SCS with progressive loss of efficacy in pain control. The patient’s cohort includes 24/60 with complex regional pain syndrome, 24/60 with failed back pain, and 12/60 with other diagnoses (pelvic pain, radiculopathy, low back pain, peripheral neuropathy, and gonalgia). It is important to note that patients treated with SCS who showed habituation phenomena had undergone different forms of stimulation (tonic stimulation in 32 patients, Burst in 18 patients, and high-frequency stimulation in 10 patients). Most patients underwent a test phase (DRG-s) for 5 to 7 days (5 patients underwent an intraoperative test phase and were implanted). 1 patient discontinued the study due to malignancy. One patient died of unrelated causes. The insurance company did not approve the second stimulator for two patients despite having a positive trial phase. Two patients had to undergo DRG-S explantation, 21 and 28 months later respectively (34-year-old man and 26-year-old woman). Among the most relevant conclusions we take into account that they found a positive trial phase rate for DRG-S of 90% (56/60 patients), a significant decrease in opioid consumption and of course long-term improvement in pain (8.7 + −1.2 steps to 3.8 + −2.1 with 34-month follow-up). They point out that withdrawal rates of tonic SCS can reach 43% and even 73% in the long term and that new forms of stimulation (burst, high frequency) can serve to rescue tonic SCS but only in 27% of cases in the second trial phase and 14% of cases in the third trial phase [22]. They associate the superiority of DRG-S for having non-dependent inhibition mechanisms exclusively on GABA, have many lower energy requirements for greater efficiency, have a lower habituation percentage and a much better coverage of dermatomes. This study is supported by the oral presentation of Mandelberg-2022 in the 2021 Annual Meeting of the Congress of Neurological Surgeons: Austin, Texas October 16–20, 2021, of the series published by Chapman [11, 12] (Table 3).

There were two systematic reviews published in 2021 (Stelter) and 2022 (Dsouza) [13, 14]. Stelter's article is a specific systematic review for chronic pain treatment (different from SDRC) with DRG-S. The search was conducted in 2020 (December 1999—2020) 1881 studies were found and 28 studies were selected, with 354 patients. Among those 28 articles found, five studies describe the lack of efficacy of a SCS prior to the implementation of DRG-S (Hunter-2017, Chapman-2020, Chapman −2020, Hunter-2019 and Giordano-2018) [62–65]. The DSouza (2022) article included a systematic search from the beginning of the databases until March 2022 of clinical studies in patients treated with DRG-S for neuropathic pain of lower limbs. He found 149 articles and selected 40. Among these studies they mention five of them in which they describe patients who had previously been treated with SCS (Van Bussel-2018, Chapman-2017, Goebel-2018, Levy-2020, Ghosh-2021) [66–70] (Table 3).

There were six cases series found of patients with DRG-S that had previously undergone SCS (Yang-2017, Halasz-2021, Smith – 2021, Pendem-2021, Sidhu-2022 and Parekh-2022) [15–20]. The study by Yang (2017) has great clinical importance for being the first study to carry out a hybrid simulation using SCS and DRG-S. They were two patients treated for chronic pain. Patient 1: 43 -year -old woman with CRPS in MID secondary to traumatic crushing injury. She was implanted in 2014 with SCS-T (16 contact electrode) improved for three months and was implanted with DRG-S (electrodes in L3 and L4) with improvement at the end of the follow-up (8 months). Patient 2: 50-year-old woman with mirror-image CRPS in the lower limbs. She was treated from 2007 to 2012 with a SCS-t system (octrode + eon electrode at T9). Due to complete loss of efficacy, she was implanted in 2015 with a DRG-s (bilateral L3) without removing the SCS. The improvement was significant and did not require the use of both stimulation systems. The study by Halasz (2021) is a case series presented during the Congress of the European Society of Stereotaxy in the city of Marseille (oral presentation). There were Six patients implanted with DRG-S among which there were 2/6 who had a previous SCS system (low back pain). Both patients were implanted with a bilateral electrode at T12, showing significant improvement. Smith's study (2021) presents three patients who could not undergo DRG-s with the usual technique due to their surgical history and proposes a new retrograde puncture technique, from top to bottom from the contralateral side. This technique had already been proposed by Chapman and Al-Kaisy [71]. Among the three patients, there were two who had already been treated with SCS-t. Patient 1: 45-year-old woman with low back pain + chronic radiculopathy, spinal surgery (discectomy + L5-S1 arthrodesis), neuropathic pain in the left L5 and S1 spinal root territory. Initially, a trial phase of an SCS was performed, without significant improvement, which is why DRG-s was decided on in S1 (50% improvement); in a second surgical procedure, a DRG-s electrode was implanted in L5 (80% improvement). The Pendem-2021 study presents the clinical case of a 60-year-old man who suffered a traffic accident and rupture of the posterior tibial tendon, with a diagnosis of post-traumatic CRPS in the right foot and previously treated with SCS (16-contact electrode in T8) that allowed initial improvement (80% for 4 months) and then habituation. DRG-S was implanted in right L5 and S1 with 90% improvement and suspension of opioid use. The Sidhu—2022 study was presented at the International Neuromodulation Society (INS) congress in Barcelona. There are three patients previously implanted with SCS for painful neuropathy and required DRG-s. Patient 1: Chronic neuropathic pain in the right knee, treated with SCS, 5 revision surgeries of the stimulator without improvement. Patient 2: Patient with CRPS in the LII treated with SCS without improvement. Required DRG-s. Patient 3: Chronic neuropathic pain in the left hemiabdomen, treated with SCS without improvement, required DRG-s. The Parekh-2023 study (oral presentation at the 26th North American Neuromodulation Society Congress, held January 12–15, 2023 in Las Vegas, Nevada) presents the case of a 30-year-old female patient with post-laminectomy syndrome (low back pain + neuropathic pain in legs) initially treated with SCS-t (50% improvement the first year and then clinical deterioration). The SCS was removed, and a trial was done with DRG-s at T12 and bilateral S1. Improvement greater than 90%. The Chapman-2021 study presents a case series of patients with severe peripheral vascular disease treated with DRG-s. Among the 3 patients, it is noted that one of them underwent a trial phase with an SCS + DRG-s, in which an SCS was implanted despite the fact that she obtained greater improvement with the trial phase of the DRG-s. Patient 2: 55-year-old woman, type 2 diabetes and severe ulcer on the first toe of the left foot. Test phase of DRG-s in left L4 and L5 and SCS (electrode in T9 and T10). DRG-s was initially activated for 3 days, stimulation was suspended for 3 days and then SCS was used for 3 days. The improvement was greater during the days of DRG-s use, not only in the intensity of pain but also in the appearance of the skin lesion and vascularization; however, for administrative reasons, SCS was implanted (Table 3).

## Classification and description of phase 2 articles

Forties articles were selected, including: 21 prospective clinical studies (Huygen-2019, Kalleward-2019, Kalleward-2020, Levy-2019, Morgalla-2019, Chapman-2021, Sverrisdottir-2020, Lo bianco-2020, Gravius-2019, Han-2024, Sankarasubramanian-2021, Weising-2022, Eldabe-2022, Mol-2022, Piedade-2022, Piedade-2023, Chapman-2024, Schulteis-2024, Mons-2024,Rigoard-2025, Van Bussel-2018) [2, 23–41], 9 retrospective clinical studies (Hunter-2019, Verrills-2019, Martin-2020, Martin-2020, Hogedorn-2021,Hines-2022, Graca-2023, Tabatabaei-2024, Mullins-2024) [42–50], 15 case series (Falowski-2019, Hunter-2019, Skaribas-2019, Verma-2024, Yang-2024, Thalla-2024, Piedade-2024, Lee-2022, Hunter-2017, Chapman −2020, Chapman-2020, Giordano-2018, Goebel-2018, Ghosh – 2021, Chapman-2021) [51–58], two systematic literature reviews (Deer-2020, Ghorayeb-2023) [59, 60] and one consensus article (Deer-2024) [6]. The aim of phase 2 is to identify patients in these clinical studies who have been included for DRG-s implantation with a history of having been treated with SCS. [2, 23–60] (Table 3).

The 21 prospective studies (Huygen-2019, Kalleward-2019, Kalleward-2020, Levy-2019, Morgalla-2019, Chapman-2021, Sverrisdottir-2020, Lo bianco-2020, Gravius-2019, Han-2024, Sankarasubramanian-2021, Weising-2022, Eldabe-2022, Mol-2022, Piedade-2022, Piedade-2023, Chapman-2024, Schulteis-2024, Mons-2024,Rigoard-2025, Van Bussel-2018) were conducted between 2019 and 2025 [2, 23–41] and included a total of 672 patients treated with DRG-s among which only 6 patients had previously had SCS. [34, 35]. It is important to note that in the prospective Eldabe cohort [35] including 42 patients implanted with DRG-s, 5 patients required subsequent implantation of a SCS after removal of the DRG-s (Table 3). The Rigoard −2025 study was included because it implants both stimulation systems in 12 patients with pain and evaluates the best responses in a prospective protocol [2].

The 9 retrospective clinical studies (Hunter-2019, Verrillis-2019, Martin-2019, Martin-2020, Hogedon-2021, Mines-2021, Graca-2022, Tabatubai-2024, Mullins-2024) gathered a total of 513 patients implanted with DRG-s among which only 21 patients had SCS [42–50]. The study by Mullins presents a series of 18 patients synchronously implanted with SCS + DRG-s for chronic neuropathic pain. 9/18 patients preferred to continue activating both stimulation systems, 3/18 patients used only the SCS and 5/18 patients DRG-s [50] (Table 3).

The 15 cases series (Falowski-2019, Hunter-2019, Skaribas-2019, Verma-2024, Yang-2024, Thalla-2024, Piedade-2024, Lee-2022, Hunter-2017, Chapman −2020, Chapman-2020, Giordano-2018, Goebel-2018, Ghosh – 2021, Chapman-2021) gathered a total of 27 patients implanted with DRG-s among which only 5 patients had SCS [51–58]. It is important to note the clinical case of Piedade of a 45-year-old patient with persistent spinal pain syndrome (PSPS) treated synchronously with SCS + DRG-s, with a test phase that showed greater improvement with the use of both devices and with the isolated use of DRG-s [57].

### Pooled analysis

147 patients were identified in the literature as having previously had a SCS, who had previously undergone a SCS trial phase or who had an implanted and active system at the time of the study. In 31/147 patients, detailed information on clinical or therapeutic aspects related to the SCS was not included in the articles. They had a mean age of 52.22 years and the gender distribution was similar (51% women/49% men). The cause of chronic pain was most frequently reported as CRPS (37%) and PSPS (36%). It included other pathologies such as: chronic pelvic pain, radiculopathy, peripheral neuropathic pain, gonalgia, post-thoracotomy pain, post-inguinal herniorrhaphy pain, phantom limb pain and severe peripheral artery disease. The follow-up period and the analysis of the results were very varied, but it can be concluded that in most cases the use of DRG-s was indicated due to a poor clinical response to SCS despite not specifying what type of stimulation was being performed (in most cases SCS-t) or whether the therapy was salvaged with other forms of SCS (SCS-B, SCS-HF, ECAP or DTM). In most patients implanted with DRG-s the clinical result was better and the degree of patient satisfaction with the new therapy was clear. [6, 11–21, 34, 43, 48–52, 57, 61–69].

There are 6 highly compelling studies in which SCS + DRG-s (hybrid stimulation) was combined (Mullins-2024, Piedade-2024, Van Bussel −2018, Ghosh-2021, Chapman-2021, Rigoard-2025) [2, 50, 57, 65, 68, 69]. We differentiate the study by Ghosh because it is the only one that presents 4 patients who were already being treated with SCS and to whom DRG-s therapy was added. All patients preferred to keep the two devices since it allowed them a better result. The studies by Mullins (18 patients), Chapman (1 patient), Van Bussel (12 patients) and Piedade (1 patient) included a test phase with implantation of SCS electrodes and DRG-s electrodes. Specific stimulation protocols were carried out, generally using each one independently and then in combination. Each change of stimulation scheme was preceded by a washout period (between two and three days). For most of these patients the best results were observed when stimulation was combined, followed by DRG-s and finally SCS [2, 50, 57, 65, 68, 69] (Table 3).

## Discussion

The treatment of chronic pain requires new technologies that allow greater efficacy and a decrease in opioid consumption with a real improvement in quality of life [2]. The principle of using a new therapy (DRG-s) with a new target of action of neuromodulation has proven to be very useful for the treatment of chronic pain and should be integrated into the therapeutic arsenal to control suffering, but its role in the control of pain in of patients previously treated with SCS should be analyzed in a more detailed manner [2, 6,72–76,77–80].

- **Number of articles**. The number of DRG-s implanted and patients treated has had an exponential curve. This suggests that the number of published articles that support its use, especially in those cases that are considered"salvation", should be high. There is still a very limited number of addressing to the specific topic of “salvation” of patients with SCS who require a DRG-s. Within the existing studies, the information is incomplete for the majority, with very varied selection and analysis parameters. In 21% of the patients, no clinical aspect was reported. The variety of clinical indications also influences the analysis, with specific aspects not being analyzed in each one.
- **Evaluation of loss of efficacy and habituation.** The terms"loss of effectiveness"and"habituation"are very broad. The decision to use a new stimulation technology (DRG-s) in patients with"loss of efficacy"or"habituation"is based on the lack (or partial and incomplete) of improvement of pain, but on all articles found the evaluation process of the SCS prior to decision-making is not mentioned. This applies not only to implanted patients but also to those who have had negative test phases for SCS and who were offered new treatments with DRG-s. The International Neuromodulation Society clearly defines the limits related to these terms and proposes a therapeutic organizational chart that must be applied.
- **Rescue of ineffective DRG-s with new SCS technologies**. Confirming the intricacy of the subject of"salvation", publications arise in which the opposite is proposed. In our review we found some cases in which the reverse process occurred and the need to rescue ineffective DRG-s with new SCS technologies is raised [68].
- **Ineffective SCS-t.** Currently, the use of SCS allows different forms of stimulation to be combined. Some of them are more effective than traditional ones (SCS-t). Most of the studies and percentages of improvement, explantation, loss of efficacy, habituation and/or tolerance are conducted with patients undergoing SCS-t, which although we are convinced of its usefulness, is the one that presents the greatest variation in the percentages in the medium and long term.
- **Hybrid stimulation.** The multidirectional treatment schemes applied in chronic pain are widely known even from the conception of the formulation of drugs. Covering various mechanisms of action allows for promising results. The noteworthy topic of hybrid stimulation, combining SCS and DRG-s systems, emerges. It is observed that patients who previously had SCS feel more comfortable maintaining both technologies rather than eliminating the previous therapy. This also applies to notable studies that carried out combined test phases with multi-phase protocols evaluating single stimulation of each of the technologies and combined evaluation. Both are useful, with a higher percentage for DRG-s [50, 57, 65, 68, 69]

Most patients included in the loss rates of efficiency (LOE) of up to 5% in the first year, 10% the second year, and explanation rates of up to 43–73% in the long term, depend on poor indications, incomplete test phases, bad electrode position, displacement, system damage, among others. It is clear that SCS cannot serve as a treatment for all types of pain such as: neuropathic pain of central origin, nociceptive pain, mixed pain, visceral pain, among others, which implies that, although we dominate the implementation technique we cannot apply it in all pain conditions [6]. In the same way we must not forget the universal premise related to neuromodulation which is to always ensure a precise etiological diagnosis that identifies an anatomical modification that causes symptoms. If it is possible to treat and improve that anatomical condition, it should always be done prior to any implementation of a stimulation system, such as: Syringomyelia in subsequent neuropathic pain through raquimedular trauma. The salvage of a SCS with a DRG-S must be analyzed independently in several different clinical situations:

- **Salvage therapy in patients with negative SCS test phase**. A primary element in the therapeutic process for chronic pain with SCS is the test phase. It is not easy to determine that it is a well -made test phase. The high percentages of SCS efficiency loss are due to poorly made or poorly analyzed test phases. Therefore, it is crucial, to establish specific parameters to determine what truly constitutes a negative test phase, but more importantly, when it is positive, as this is what will justify an intervention and could be the reason that, years later, we speak of the need for salvation. The stimulation trial phase must be performed mandatorily before implantation [76–78, [79–81]. The electrode should be placed according to Chipault's law in the spinal segment corresponding to the pain area and verified with an intraoperative test to ensure the perception of paresthesias covering the affected area [4]. The evaluation time for the trial phase should be as long as necessary based on the clinical response, without being overly optimistic but also not withdrawing it 24 h after starting the process due to lack of efficacy. Ideally, the improvement obtained after a trial phase should be greater than 50% and preferably over 75%, considering that it involves a high-cost resource. Employing permanent electrodes during the trial phase limits the objectivity of the analysis. Using trial electrodes and removing them at the end of the trial phase allows the patient to have a washout period from the stimulation and feel their pain again once the stimulation is suspended, confirming the system's usefulness. Only after a proper analysis of the indication and a strict trial phase, which includes prior evaluation and identification of risk factors for a poor response to treatment, is a permanent implant decided. Ideally, using systems that offer the greatest number of stimulation options. Only after meeting these requirements can objective conclusions be drawn [4, 6].
- **Salvage therapy in patients with implanted SCS who have experienced loss of efficacy, tolerance, or habituation.** In these patients, It is important to detail the clinical response and assess the characteristics of the pain. It is not enough to establish a simple percentage of improvement and a number on the pain scale. Under such conditions, The following aspects should be considered: ruling out the emergence of a new pathology or a modification of the previous one, which requires a specific diagnostic and therapeutic analysis. A patient with SCS may develop pain of a different origin over time and is not necessarily limited to symptoms of their initial condition. It is important to rule out electrode displacement or damage to the stimulation system. X-rays of the system should be taken to verify the position and integrity of the wires. Impedance should be measured to confirm proper current flow. This type of patient has a better prognosis regarding their response to new forms of SCS. They respond better to a combination of SCS-B, SCS-HF programs, and even the most advanced technologies. Salvage therapy in these patients may involve repositioning the electrode, modifying the pulse generator to offer new technologies, or treating the specific pathology (blocks, radiofrequency, surgery) of the new condition.
- **Salvage therapy in patients with implanted SCS who experience no clinical improvement, or only partial or incomplete improvement (from the start of treatment)**These patients have a worse prognosis regarding their recovery. It is important to always consider that this therapy may not have been ideal from the start, and the cause could be a poor indication, an improperly conducted trial phase, or a poor surgical decision regarding the electrode placement site. Other possible causes of pain and their specific treatments should be explored [80, 82]. The proper position of the electrode should be verified with system X-rays, as well as the integrity of the current flow and the use of other forms of stimulation. It will always be necessary to optimize the SCS and even consider a virtual explantation before deciding to place a DRG-s. In cases of incomplete improvement, there may be room for hybrid stimulation (SCS + DRG-s), but we believe it is not advisable.
- **Salvage therapy in a patient with an implanted DRG-s without improvement.** The considerations related to SCS also apply to patients with DRG-s who show loss of efficacy or lack of clinical response. There are already articles showing patients who required changes in the stimulation system to salvage therapy, switching from DRG-s to SCS. This aspect is highly pertinent as we see more and more articles demonstrating the utility of DRG-s in a wide range of very chronic pain indications, including nociceptive pain, visceral pain, and mixed pains, where neuromodulation typically has limitations. We should be optimistic about the effect of electricity in pain control, but the real utility must be properly evaluated. These considerations also apply to new peripheral nerve neuromodulation techniques [50, 57, 65, 68, 69, 83].

## Conclusions

DRG-s emerges as a potential treatment option in patients with chronic pain who have been previously treated with SCS. It is advisable to follow the international guidelines to evaluate habituation or loss of efficacy phenomena in patients with SCS before considering DRG-s as the first option of"salvation". More prospective clinical studies are needed to evaluate the long-term outcome of DRG-s as"salvation"in patients with chronic pain previously treated with SCS.

## Data Availability

No datasets were generated or analysed during the current study.
